# Preload loss of abutment screws after dynamic fatigue in single implant-supported restorations. A systematic review

**DOI:** 10.4317/jced.54374

**Published:** 2017-11-01

**Authors:** Beatriz Pardal-Peláez, Javier Montero

**Affiliations:** 1 DDS. PhD in Dentistry. Graduate in Odontology. Postgraduate Student of the Department of Surgery. University of Salamanca. Campus Miguel de Unamuno. Salamanca, Spain; 2 DDS. PhD in Dentistry. Graduate in Odontology. Tenured Lecturer in Prosthodontics of School of Dentistry. University of Salamanca. Campus Miguel de Unamuno. Salamanca, Spain

## Abstract

**Background:**

To carry out a systematic literature review of the causes of preload loss of the abutment screws, of internal and external connection implants, tightened to different torque values and subjected to cyclic loading.

**Material and Methods:**

A systematic search was conducted in PubMed, EMBASE, and Cochrane Library databases with reference to *in vitro* studies in which internal and external connection implants were subjected to cyclic loads to determine the degree of loosening of the abutment screws after loading.

**Results:**

The reviewed studies tested distinct implant connections (mostly externally hexed, and morse taper) subjected to diverse cycles (from 16667 to 1 million), with loads ranging from 0-400 Nw, using screws of different materials and designs that were tightened into torques between 20-45 Ncm, Accordingly after loading the percentage of torque loss ranges between 16.1% to 39%.

**Conclusions:**

Most of the studies indicate that the internal connection, together with the morse taper, best resists cyclic loading in terms of screw loosening in single-tooth implants.

** Key words:**Dental Implants, Dental Implant-Abutment Design, Torque, In Vitro Techniques, Systematic Review.

## Introduction

For many years now, the loss of natural teeth has created a need for tooth replacement for both aesthetic and functional reasons. In 1978, Brånemark and Albrektsson presented, at Harvard University, the results of their fifteen-year-long investigation concerning the integration of titanium in bones, at which time they coined the terms ‘osseointegration’ and ‘the implant-prosthetic complex’ (1). The latter refers to an osseointegrated implant whose connection can either be internal or external. The implant comprises a prosthetic abutment, over which the crown is placed, and a screw that joins the abutment to the implant (1-3).

Initially, osseointegrated implants presented a high success rate (being 84% in the maxilla and 93% in the mandible during a 5-12 year observation period (4); however, soon after, relatively high levels of loosening of the screw abutment were observed (12.7% at 5 years) (5,6). This loosening is one of the main problems associated with prosthetic implants (5,6). Moreover, it has been shown that 43% of abutment screws become loose during the first year of placement (7), and that the cause of this loosening can be due to either the incorrect biomechanical design of the interface or occlusal overload (8). Also, if the loosening process continues over a long period of time it could lead to screw fracture (0.35% at 5 years) (5,6).

The first dental implants were comprised of an external connection system (0.7mm-high hexagon), with internal connection implants appearing later (8). In the external connection, the hexagonal anti-rotational component is the most frequently used; however, the rate of loosening with this type of connection has been shown in the literature to be between 6 and 48%5. In the case of the internal connection, internal hexagon or octagon are used and allow for a more exact union between the implant and the abutment, which in turn reduces the movement of the interface and in principle decreases screw loosening (9,10). Another option for an internal connection is the morse taper that introduces an internal cone of 8° or 11° (11,12). It has been proposed that the morse taper joint could protect against screw loosening (11,12).

The osseointegrated implant and the prosthetic abutment are joined together by a screw, and have therefore been called a screw joint (13).

Abutment screw stability can be affected by preload, the effect of settling, and screw geometry (13,14). Preload is the force, measured in volts and later transferred to newton, that is generated when a screw is tightened within a given torque (13,15). Only 10% of the initial torque is transformed into preload, where the remaining 90% is used to overcome the friction between the surface irregularities (13,15). Another important phenomenon experienced by the screw joint is the settling effect. This occurs because neither the interior torque nor the screw is perfectly fabricated without irregularity, and therefore these rough areas are smoothed out causing a loss of 2-10% of the initial preload (13). It is known that the preload should not be too high and should be lower than 75-80% of the elastic limit of the material (13,15). If the forces applied onto the system are greater than the preload, screw loosening takes place (13,15).

From a clinical point of view, it is thought that screw loosening is greater in an external connection than in an internal connection, where the incidence of loose screws is 38% in systems with an external hexogon (7,16). However, there are no qualitative data comparing loosening between external and internal connections.

Torque loosening causes micromovements in the interface to appear that generate both mechanical problems (increased loosening and failure of the screw, abutment and implant body) and biological problems. In the case of biological problems, microspaces that form within the interface permit the colonization of bacteria that can cause mucositis, peri-implantitis and finally implant loss, especially when the implant-prosthesis are subjected to cyclic loads (17).

The clinician should be aware, when selecting the type of implant and torque to be applied, that the abutment screw can be influenced in terms of the biomechanical yield of the implant-prosthesis.

Despite the number of existing *in vitro* studies related to the loosening of abutment fixation screws subjected to cyclic loads, in actual fact, there are only a few publications which compare the effect of the connection and the effect of the applied torque in the loss of preload in the presence of repeated occlusal loads. This work aims to review in a systematic way the existing literature regarding the conditioning factors of preload loss of the abutment fixation screws, in internal- and external-connection implants, tightened to varying torque values and subjected to cyclic loads.

## Material and Methods

A literature search was conducted regarding articles, written in English, from 1995 to 2016 in relation to *in vitro* studies where dental implant units, with either internal or external connection, were subjected to cyclic loads to compare the degree of loosening of the abutment fixation screws using the measurement of counter torque.

To do so, the PubMed, EMBASE, and Cochrane Library databases were searched with different search equations (see  Table 1), using keywords (screw, mechanics, implants…) and free terms (screw loosening).  Table 1 shows how the conceptual terms or keywords were automatically included into the headings to carry out the search with free terms.

**Table 1:**
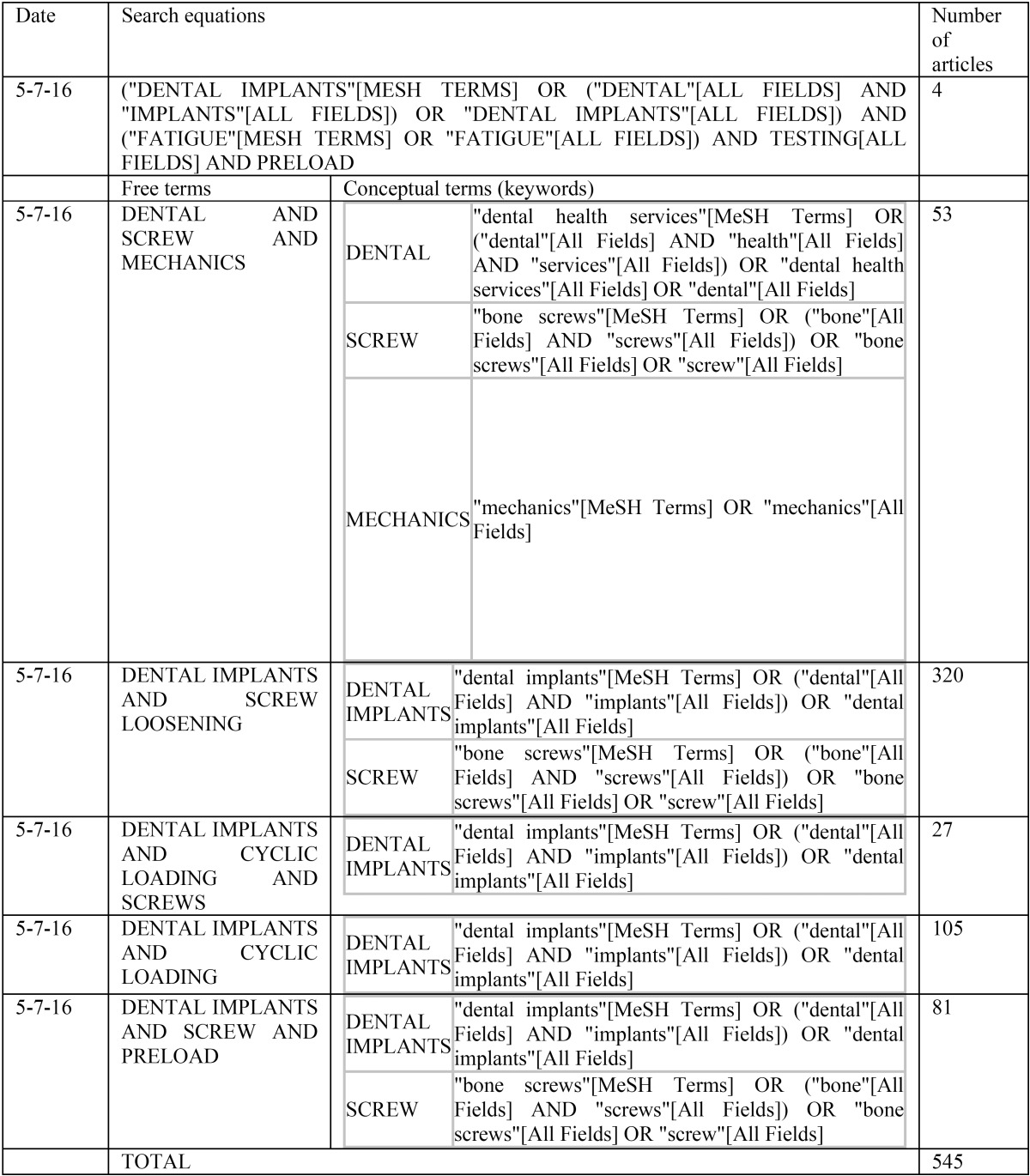
Search strategies and the number of inputs used in the literature review.

**Figure 1:**
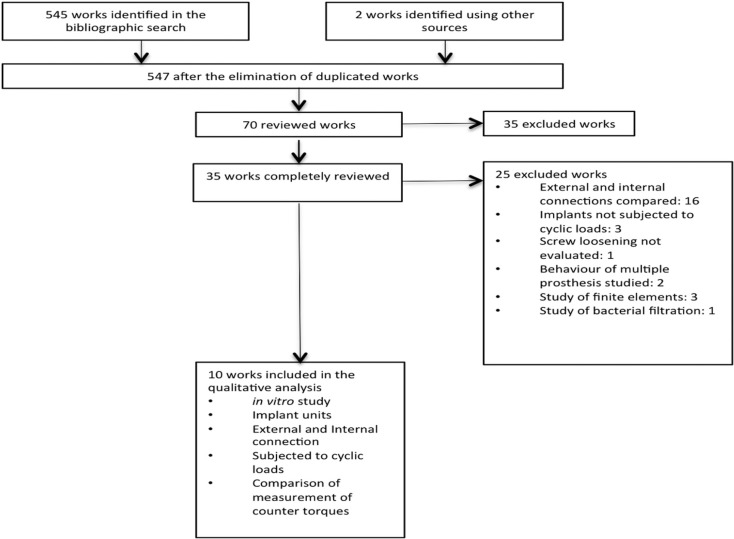
PRISMA diagram of the selected articles.

The following types of work were excluded from the search: those that did not specify the type of connection used, those whose objective was not to compare internal and external connections, where the implants were not subjected to cyclic loads, those that studied the behaviour of multiple prostheses or only bacterial filtration, and those where the analysis was based on finite elements. The search resulted in the identification of a total of 545 papers, after 70 studies were eliminated due to being duplicated. The abstracts of all 70 articles were reviewed, and 35 of these were selected and fully read. The other 35 articles were discarded since it was determined after reading the abstract that they did not comply with the objective of this work. Finally, 10 articles that fulfilled the criteria of inclusion were selected, as shown in the diagram (Fig. 1). Therefore, the final article sample was made up of 10 *in vitro* studies, in which implant units with internal and external connections had been subjected to cyclic loads, and the degree of the loosening of the abutment fixation screw was subsequently analysed using the measurement of counter torque.

## Results

As shown in  Table 2, there is a great variety of methodological approaches used amongst the different studies.

**Table 2:**
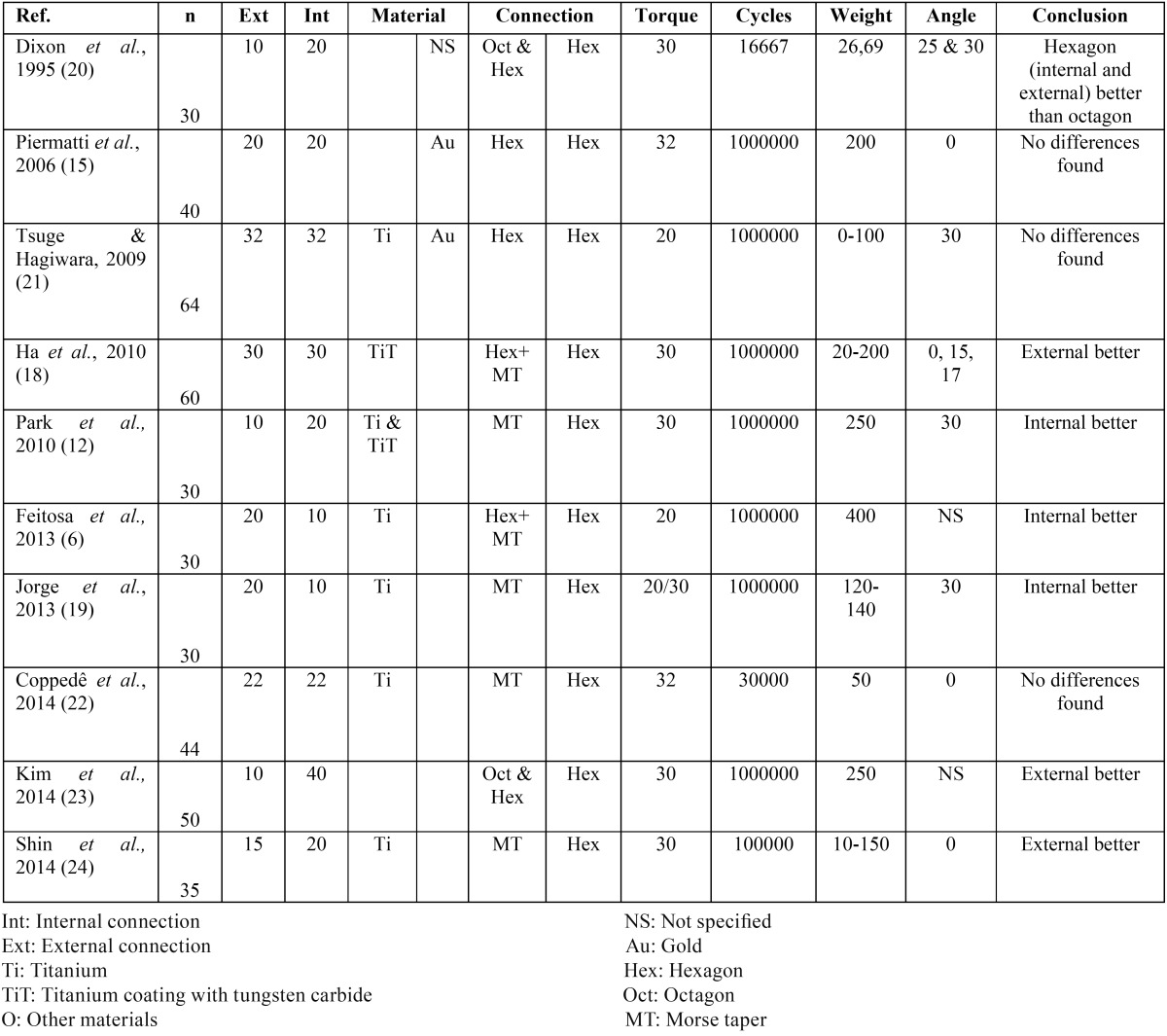
Main characteristics and findings of the reviewed works.

In all of the works, except for Park *et al.* (12), the screws were first exposed to a sequence of tightening and loosening, before being subjected to cyclic loads, in order to determine the removal torque value, which was later compared to the counter torque value after the mechanical cycle.

The applied screw and removal torque values were measured, after loading, using a previously calibrated torquemeter, giving a torque value in newton per centimetre (Ncm). In addition, some authors used a thermo-cycler to reproduce the oral environmental conditions with greater accuracy (18,20).

Most of the studies reviewed, set the implants over an acrylic resin with an elastic modulus of 17 GPa, similar to that of bone (6,9,15,19,20). However, Tsuge and Hagiwara (21) set the implants over aluminium with an elastic modulus of 70 GPa, much greater than the elastic modulus of human bone. Coppedê *et al.* (22), used stainless steel (elastic modulus of 190 to 210 GPa) and other authors placed the implants in metal supports; although, the type of metal used was not specified (12,18).

The sample size ranged between 30 (6,12,19,20) and 64 implants (21). The type of connections used were universal hexagon for the external connection, and the internal octagon (9,20,23), hexagon (6,18,20,23), or morse taper (6,12,20,22,24), or a combination of morse taper with an antirotational hexagon (18), for internal connection.

Regarding the screw material, the majority of the works used titanium alloys coated with or without tungsten carbide (6,12,18,20,22,24,25), or with gold. Another study compared these two types of coating (20). Dixon *et al.* (20) do not specify which type of material are the screws employed in their study made of, and Tsuge and Hagiwara (21) stated that torque maintenance was better with screws made of titanium alloys than with those made of gold, regardless of if they were subjected to loads or not.

The design of the screws was not specified in the majority of the studies. However, Coppedê *et al.* (22) compared the performance of flat-head screws to those with a conical head, and concluded that the conical-head screws produced better results. The work carried out by Piermatti *et al.* (15) reported that long and conventional flat-head screws with a machined journal were better, and highlighted the importance of screw design in preload maintenance.

Furthermore, some authors utilized different types of abutments in their studies, which added a new variable, abutment type, to the comparison of torque loss between implants with internal and external connections. Dixon *et al.* (20), compared the use of straight and angled abutments and arrived at the conclusion that there were no differences between either types with respect to screw loosening. They also concluded that implants with an internal hexagon connection exhibited the greatest number of differences between both types of abutments regarding the torque required to loosen them. However, these types of connections were similar, or even better than those implants with an external or internal octagon. Dixon *et al.* (20) also observed that preload maintenance was not different between internal and external connection when the anti-rotational component was a hexagon, and that the performance of the joint was worse in implants with an internal connection with an internal octagon. Ha *et al.* (18) compared different types of abutments in internal and external connection implants, and the performance was statistically significantly better for implants with an external connection, where angled abutments had much better counter torque values than straight abutments. Moreover, Jorge *et al.* (20) reported a better performance for implants with an internal morse connection (with a percentage of torque loss of 32.88%) than those with an external connection, independently of whether the abutment used was conical or UCLA, and had a significantly better performance when compared to an implant with an external connection and a UCLA abutment (percentage of toque loss of 39%).

## Discussion

When determining the most important factors that condition screw abutment loosening in the prosthetic implant complex, it is problematic to use *in vivo* comparison studies as this type of analysis concentrates on a great number of prosthetic factors that are difficult to control. Therefore, this work has dealt with *in vitro* studies in which the biomechanical performance of abutment fixation screws in internal and external connection implants, made of different materials and torque values, has been compared and focuses on the loss of preload as an indicator of the risk of loosening.

Prosthetic abutment screw loosening is one of the most prevalent complications associated with dental implants. The percentage of torque loss reported in the literature ranges between 16.1% and 25% (20); however, the work by Jorge *et al.* (20) discovered a slightly higher loosening rate that ranged from 19.7% to 39.0%.

There are a great number of *in vitro* studies which specify that the performance of the internal connection is better than the external connection, as supported by the published works of Park et al., Jorge *et al.* and Feitosa *et al.* (6,12,20). In contrast, some of the reviewed studies came to the conclusion that the external hexagon connection has the best torque maintenance with regards to screws (18,23,24). Lastly, some authors have stated that the connection design is not a determining factor of screw loosening, but is dependent on other factors such as the material and the design of the screw and the type of abutment (15,21,22).

Such diverse results may be explained by the type of methodology employed, because although it is similar in all of the works reviewed, there are some slight variations. The number of cycles applied ranges from 16667 to one million, the weight ranges from 0 N to 400 N, and the use of screws of different materials and designs that are tightened into torques varying between 20 Ncm and 45 Ncm, are as shown in  Table 2.

As a result of the literature review, five factors have been identified by the authors as being important regarding the torque loss of screws: the type of connection, the design and material of the screw, the type of prosthetic abutment, the settling effect, and internal loads.

Type of connection

Most of the authors of the reviewed works agreed that the morse taper was more effective at maintaining the preload than the external hexagon connection (12), and in some cases, more effective than the internal hexagon connection (6). This may be due to the fact that in the external connection the axial loads occur directly over the screw. By contrast, in the internal connection, the forces are transferred more deeply, making the system more stable. In an internal connection with a morse taper, it is the reduction of micromovements that increase stability (18) and improve the dissipation of stress (20). Therefore, the use of internal connection implants would be most suitable for single prostheses, while the use of the external connection would be reserved for multiple prostheses (9).

-Material and screw design

There seems to be a lack of consensus with respect to the appropriateness of coating the screw with some type of dry lubricant in order to maintain preload. Although, some authors have found that screw coatings, such as TorqTiteTM (TorqTiteTM, Nobel Biocare UK Ltd, County Wicklow, Ireland) and Gold-Tite® (Gold-Tite®, 3i Implant Innovations, Inc. West Palm Beach, FL, USA) (6), do improve performance, as compared to those made of gold or titanium alloy without any type of coating (18,25). In contrast, Tsuge and Hagiwara (21) concluded that titanium alloy screws were less prone to loosening than the Gold-Tite® screws, regardless of the type of connection. Regarding screw design, Piermatti *et al.* (15) determined, after comparing the performance of four different brands of implants subjected to cyclic loads, that screw design was the most important factor in the stability of the prosthetic implant. They also determined that screws with a long rod and a machined journal were better at maintaining the preload, and that the connection design was not a significant factor for torque loss. Coppedê *et al.* (22) concluded that conical-head screws were less likely to come loose than those with a conventional flat head.

Therefore, the material and/or the design of the screws seem to have an influence on preload maintenance, in which a gold screws (18,25) with a long rod, a machined tip (15) and a conical head (22) give the best performance. In contrast to what was originally thought, there does not seem to be a correlation between prosthetic vertical imbalance and torque loss (20). Regarding the diameter of the screw joint, it appears that a larger diameter is better in terms of torque maintenance (20).

-Settling effect and cyclical loads

The main cause of screw loosening is the settling effect, which explains why all screws initially suffer from a preload loss of between 2% to 10% (7), without being subjected to any type of load. Because of this, some authors have recommended that screws be retightened 10 minutes after the first tightening, and again after being subjected to cyclic loads (13,24). Hence, the initial settling is eliminated, and the preload is recovered.

Siamos *et al.* (13) found that retightening the screws after 10 minutes reduced the percentage of torque loss by 17-19%. It seems, after reviewing the various articles included within this study, that retightening acts as a positive factor in maintaining preload (6,12,20-24).

All of the works reviewed, except for the study by Tsuge and Hagiwara (21), showed that the screws were less loose at baseline, before applying any load, than after receiving the cyclic loads (12,15,18,19,24), regardless of the type of connection, screw or abutment. However, this difference was statistically significantly in a only a few of the studies (22,23). Screw loosening was associated with the occurrence of micromovements in the interface when the screw was exposed to external loads, which subsequently increased torque loss (19).

Tsuge and Hagiwara (21) explain the lack of concordance of their results with the rest of the other published works to the possibility of a lack of force transfer to the screw, or the deterioration of the adhesive contact surfaces that leads to an optimal plastic deformation of the screw which in turn improves the overall preload maintenance after application of the cyclic loads.

Although the reviewed works show internal validity and use a reproducible method, there is still a lack of a standardized methodology in the application of the cyclic loads to the implants. Park et al. (12) followed the ISO 14801 (26) standard in their methodology, which is specific to testing implants, and subjected them to 5 million load cycles. However, this norm does not require a standardized type of torque and is therefore specific for each type of connection and screw. Hence, the comparisons of the bio-mechanical performance reported in this study are not univariant and are subject to confounding bias.

As summary, in the reviewed literature, there seems to exist a certain level of consensus that establishes that the internal connection, together with the morse taper, is the type of connection that is the most resistant to cyclical fatigue in terms of screw loosening in single-tooth implants. However, screw loosening is a multifactorial event that depends, not only in the type of connection, but also in screw design and material, type and design of abutments, passive fit of the prosthetic elements and occlusal forces among others.
